# Supplementary material for: Determinants of post-acute COVID-19 syndrome among hospitalized severe COVID-19 patients: A 2-year follow-up study
Source: PLoS One. 2024 May 10;19(5):e0298409. doi: 10.1371/journal.pone.0298409 (PMC11086844; doi:10.1371/journal.pone.0298409)
Supplement: Attachment — Submitted filename: Response to Reviewers.docx [file pone.0298409.s002.docx]

PONE-D-23-27462R1

Assessment of the prevalence of post-acute COVID-19 syndrome and its associated risk factors among severe COVID-19 patients admitted to Millennium COVID-19 Care Center, Addis Ababa, Ethiopia

*Dear Editor and Reviewers;*

*I hope this letter finds you well. I would like to express my gratitude for taking the time to review my manuscript. Your constructive feedback and comments have been invaluable in improving the quality and clarity of my work.*

*I would like to address the concerns and comments raised in your review. I have carefully considered each one of them and have made the necessary changes to the manuscript accordingly.*

*So please find the following comments and responses.*

Journal Requirements:

When submitting your revision, we need you to address these additional requirements.

1. Please review your reference list to ensure that it is complete and correct.

-	*I've re-checked the reference list and found it to be complete and correct.*

2. If you have cited papers that have been retracted, please include the rationale for doing so in the manuscript text, or remove these references and replace them with relevant current references.

- *No retracted papers are cited in the current submission.*

3. Any changes to the reference list should be mentioned in the rebuttal letter that accompanies your revised manuscript. If you need to cite a retracted article, indicate the article’s retracted status in the References list and also include a citation and full reference for the retraction notice.

- *No changes are made to the reference list.*

Additional Editor Comments:

Please revise and shorten the Title.
There is no need to specify the city name in the Title unless necessary.

*Thank you for the comment. The title is now revised to “Determinants of Post-acute COVID-19 Syndrome among hospitalized severe COVID-19 Patients: A 2-year follow-up study”*

Reviewers' comments:

Reviewer's Responses to Questions

**Comments to the Author**

1. If the authors have adequately addressed your comments raised in a previous round of review and you feel that this manuscript is now acceptable for publication, you may indicate that here to bypass the “Comments to the Author” section, enter your conflict of interest statement in the “Confidential to Editor” section, and submit your "Accept" recommendation.

Reviewer #1: All comments have been addressed

Reviewer #2: All comments have been addressed

- *Thank you for the positive response.*

2. Is the manuscript technically sound, and do the data support the conclusions?

The manuscript must describe a technically sound piece of scientific research with data that supports the conclusions. Experiments must have been conducted rigorously, with appropriate controls, replication, and sample sizes. The conclusions must be drawn appropriately based on the data presented.

Reviewer #1: Yes

Reviewer #2: Yes

- *Thank you for the positive response.*

3. Has the statistical analysis been performed appropriately and rigorously?

Reviewer #1: Yes

Reviewer #2: Yes

- *Thank you for the positive response.*

4. Have the authors made all data underlying the findings in their manuscript fully available?

The [PLOS Data policy](http://www.plosone.org/static/policies.action#sharing) requires authors to make all data underlying the findings described in their manuscript fully available without restriction, with rare exception (please refer to the Data Availability Statement in the manuscript PDF file). The data should be provided as part of the manuscript or its supporting information, or deposited to a public repository. For example, in addition to summary statistics, the data points behind means, medians and variance measures should be available. If there are restrictions on publicly sharing data—e.g. participant privacy or use of data from a third party—those must be specified.

Reviewer #1: Yes

Reviewer #2: Yes

- *Thank you for the positive response.*

5. Is the manuscript presented in an intelligible fashion and written in standard English?

PLOS ONE does not copyedit accepted manuscripts, so the language in submitted articles must be clear, correct, and unambiguous. Any typographical or grammatical errors should be corrected at revision, so please note any specific errors here.

Reviewer #1: Yes

Reviewer #2: Yes

- *Thank you for the positive response.*

6. Review Comments to the Author

Please use the space provided to explain your answers to the questions above. You may also include additional comments for the author, including concerns about dual publication, research ethics, or publication ethics. (Please upload your review as an attachment if it exceeds 20,000 characters)

Reviewer #1: The authors have addressed all the issues raised by the reviewers and current iteration merit publication after some editorial polishing of the paper. Good job

Reviewer #2: (No Response)

- *Thank you for the positive response.*
